# A Tense Race: Correlation of Liver Stiffness with Ultrasound Elastography and Hemodynamics in Fontan Patients

**DOI:** 10.32604/chd.2025.065661

**Published:** 2025-04-30

**Authors:** Yuen Lo Yau, Matthew S. Purlee, Lindsey M. Brinkley, Dipankar Gupta, David M. Saulino, Dalia Lopez-Colon, John-Anthony Coppola, Dhanashree Rajderkar, Himesh Vyas

**Affiliations:** 1 Congenital Heart Center, Department of Pediatrics, College of Medicine, University of Florida, Gainesville, FL 32610, USA; 2 College of Medicine, University of Florida, Gainesville, FL 32610, USA; 3 Department of Pathology, Immunology, and Laboratory Medicine, College of Medicine, University of Florida, Gainesville, FL 32610, USA; 4 Department of Radiology, Division of Pediatric Radiology and Nuclear Medicine, College of Medicine, University of Florida, Gainesville, FL 32610, USA

**Keywords:** Fontan, Fontan-associated liver disease, ultrasound elastography

## Abstract

**Background:**

Patients with Fontan physiology are predisposed to congestive hepatopathy, progressive liver fibrosis, and end-stage liver disease. Ultrasound-based shear wave elastography (SWE) is a non-invasive tool to diagnose and monitor liver fibrosis. We sought to determine whether the degree of hemodynamic derangement prior to and after the Fontan operation is associated with increased liver stiffness measured by SWE.

**Methods:**

A single-center retrospective study of patients with Fontan circulation who underwent ultrasound elastography between 2008 and 2024 was conducted. Liver stiffness was measured by SWE and reported as velocity in m/s. Hemodynamic assessment with cardiac catheterization and echocardiograms were collected before and after the Fontan operation. Data was analyzed as a continuous (linear regression model) and a dichotomous variable (*t*-test).

**Results:**

78 post-Fontan ultrasound elastography studies performed in 56 patients were analyzed. Pre-Fontan hemodynamics included median effective pulmonary flow of 2.45 L/min/m^2^ [2.21, 3.16] (*p* = 0.46), ventricular end-diastolic pressure of 10 mmHg [8, 10.5] (*p* = 0.77), and median Glenn pressure of 12 mmHg [13, 15] (*p* = 0.83). Post-Fontan median systemic cardiac index was 2.80 L/min/m^2^ [2.4, 3.34] (*p* = 0.93), median ventricular end-diastolic pressure of 12 mmHg [13.5, 14] (*p* = 0.99), median systemic saturation of 93% [87, 96] (*p* = 0.77), median indexed pulmonary vascular resistance of 1.80 WU·m^2^ [1.49, 2.37] (*p* = 0.93), and median Fontan pressure of 18 mmHg [16, 21] (*p* = 0.86). No correlation was found between SWE and hemodynamics. On echocardiography, no correlation was found between SWE and systemic ventricular systolic function (*p* = 0.35) or degree of systemic atrioventricular valve regurgitation (*p* = 0.35).

**Conclusions:**

The degree of liver stiffness by SWE in this cohort did not correlate with pre- and post-Fontan hemodynamics on cardiac catheterization, degree of ventricular dysfunction, or severity of atrioventricular valve regurgitation by echocardiography.

## 1 Introduction

Survival in patients after the Fontan operation continues to improve with advances in surgical techniques and medical care. The current death or transplant-free survival rate is estimated at 69–84.6% at 30 years, depending on the type of Fontan [1,2]. As a result, comorbidities associated with this non-physiological cardiac circulation are increasingly being diagnosed [3]. One of the most common complications encountered is Fontan-associated liver disease (FALD). This manifests as a combination of structural (liver congestion, fibrosis, cirrhosis, nodules, and hepatocellular carcinoma) and functional derangements (elevated liver enzymes or bilirubin levels, altered synthetic function), with a broad spectrum of severity [4].

The main mechanisms contributing to the development of FALD are chronic elevation of central venous pressure (leading to congestive hepatopathy with increased sinusoidal pressure) and reduced cardiac output (causing hepatic ischemia due to compromised arterial and portal circulation). Elevated inflammatory mediators and neurohormonal activation are associated with FALD [5]. Elevated biomarkers such as interleukin 6, growth-derived factor-15, tumor necrosis factor α, and β2-macroglobulin have also been described in Fontan patients [6].

While FALD is considered a ubiquitous complication of the Fontan procedure, the evaluation of liver disease in this population has not been standardized. Liver biopsy remains the gold standard for grading the degree of fibrosis [7]. Unfortunately, this entails an invasive procedure that can be performed by transvenous or percutaneous access with rare but severe adverse events as well as the risks of sedation or anesthesia [8]. Therefore, liver biopsy is not ideally suited as a serial surveillance tool. Non-invasive studies such as liver function tests and imaging, including liver ultrasound (US), magnetic resonance imaging (MRI), elastography (by US or MRI), and computed tomography (CT) scan, have all been used with variable utility. Given the lower cost, lack of ionizing radiation, and widespread availability of ultrasound technology, elastography has been adopted as a standard screening tool in this population [9].

Two types of elastography are used: strain and shear-wave elastography. In this study, we utilized shear-wave elastography (SWE) measured in m/s, with increasing SWE values associated with increasing liver stiffness [10]. Although this is a promising tool, correlation with liver biopsy to assess FALD has been controversial, as patients with Fontan physiology may have abnormal liver elastography due to vascular congestion [3,11]. If that were the case, one could hypothesize that patients with worse hemodynamics would have worse elastography results. We sought to assess whether hemodynamics before and after the Fontan operation are associated with increased liver stiffness measured by shear-wave elastography (SWE).

## 2 Methods

1. **Patients:** Our study was a single-center, retrospective study. The institutional database was used to identify patients with Fontan palliation who were followed at our center, irrespective of the site where the operation was performed. We selected patients who underwent evaluation of liver stiffness with US elastography between January 2008 and May 2024. The Institutional Review Board of University of Florida College of Medicine, USA approved this study with a waiver of consent (IRB202301611).
2. **Hemodynamic assessment:** Cardiac catheterization data before and after the Fontan operation systemic cardiac index (Qs), Fontan pressure, systemic ventricle end-diastolic pressure, indexed pulmonary vascular resistance, and systemic saturation.
3. **Echocardiogram:** The echocardiogram performed at our center closest to the date of US elastography was reviewed. The degree of systolic function and atrioventricular valve regurgitation was classified as “better than moderate” or “moderate or worse”.
4. **US elastography:** Elastography was performed by the radiology department at our institution. The Phillip EPIQ 5 system with C5-1 probe is used. Per our standard protocol, patients were fasting for 4 h before the study. Ten measurements are made on the right lobe of the liver (location is usually 1.5 cm below the capsule and avoiding major vessels). Patients were instructed to hold their breath on exhalation whenever possible. Patients held their breath ideally for a minimum of 6–8 s while the cine acquisition was obtained. Liver stiffness was reported as SWE velocity in m/s.
5. **Analysis:** Data was analyzed as continuous (linear regression model) and as a dichotomous variable (*t*-test), with *p* values ≤ 0.05 considered statistically significant.

## 3 Results

Seventy-eight US elastography studies performed in fifty-six Fontan patients were analyzed. Patient demographics are summarized in Table 1. The median age at US elastography evaluation was 12.5 years [9, 16.75], with 37 patients (66 %) being under 18 years of age. The most common diagnosis was hypoplastic left heart syndrome (*n* = 25, 44.6%), with the most common type of Fontan operation being extracardiac conduit (*n* = 44, 78.6%), and most patients did not undergo a Fontan fenestration (*n* = 31, 55.4%). The most common non-liver-related comorbidities were atrial arrhythmia (*n* = 21, 55.3%) and thrombocytopenia (16%, 42.1%). On follow-up, 10 patients (17.8%) underwent isolated heart transplant, and one patient (1.8%) underwent a combined heart and liver transplant. All data analyzed were from studies performed before the patient underwent transplantation.

The median time between Fontan operation and US elastography was 9 years [5, 14]. The median SWE in all patients was 1.96 m/s [1.65, 2.01]. We did not find a significant difference between SWE and time (years) from Fontan operation (*p* = 0.927). SWE as a function of time from Fontan operation (<5 yr, 5–10 yrs, >10 yrs) did not vary significantly (Table 2).

Hemodynamic assessment included cardiac catheterization pre- and post-Fontan operation and post-Fontan echocardiogram. Cardiac catheterization and SWE on US elastography were analyzed as continuous and dichotomized variables (Table 3 and Table 4). Thirty-five patients had adequate pre-Fontan cardiac catheterization data available. The median Qep was 2.45 L/min/m^2^ [2.21, 3.16], median systemic ventricular end-diastolic pressure was 10 mmHg [8, 10.5], and median Glenn pressure was 12 mmHg [13, 15]. No correlation was found between pre-Fontan hemodynamics and SWE velocity (*p* > 0.05).

Thirty-seven patients had post-Fontan cardiac catheterization data available for analysis. The median Qs median was 2.80 L/min/m^2^ [2.4, 3.34], median systemic ventricular end-diastolic pressure was 12 mmHg [13.5, 14], median Fontan pressure was 18 mmHg [16, 21], median systemic saturation was 93% [87, 96] and median indexed pulmonary vasculature resistance was 1.80 [1.49, 2 .37]. No correlation was seen between the above post-Fontan hemodynamics and SWE velocity (*p* > 0.05).

There was no correlation between the degree of systemic ventricular dysfunction or the severity of atrioventricular valve regurgitation on echocardiogram and SWE velocity (*p* > 0.05) (Table 5).

## 4 Discussion

Introduction of the Fontan operation by Fontan and Baudet in 1968 [12] and by Kreutzer et al. in 1971 [13] was a groundbreaking step in managing patients with a single ventricle. However, the Fontan operation is considered a palliative procedure given the absence of a mechanical pump to support the pulmonary circulation. Fontan circulation may result in significant long-term complications. Surveillance for these complications, early diagnosis, and appropriate treatment are crucial to improve patient outcomes.

FALD evaluation and prevention have gained the attention of those taking care of patients with Fontan circulation, mainly due to the long-term implications and the possible need for a heart or a combined heart and liver transplant in this population. This task remains challenging as liver biopsy, an invasive test, is the current gold standard for diagnosis and staging of liver disease.

Although US elastography has been used in other populations, there is limited data in patients with Fontan circulation [14]. With the hypothesis that elevated central venous pressure leads to increased liver stiffness, baseline SWE velocity is higher in the Fontan population. The American Heart Association scientific statement and expert consensus recommends using an upper limit of 2.2 ± 0.38 m/s in the Fontan population compared to 1.1 ± 0.29 m/s used in other disease processes [3]. For our study, we did not use the METAVIR characterization based on age for SWE [15].

Prior studies have found a correlation between liver stiffness using US elastography with age at Fontan, time since Fontan procedure, pulmonary vasculature resistance, presence of a fenestration, and systemic arterial oxygen saturation [16-18]. In prospective studies, time from Fontan operation had a weak correlation with liver stiffness, which suggests that the duration of this non-physiological circulation contributes to elevated SWE in addition to multiple other factors [19]. A study in adult Fontan patients demonstrated a correlation with the degree of liver stiffness and adverse outcomes (death, heart, heart-liver transplant, paracentesis, and worsening liver function) [20].

Sparse studies comparing pre-Fontan hemodynamics with SWE have been reported. To our knowledge, there is no data on whether adverse hemodynamics before the Fontan operation may also be associated with a higher likelihood of abnormal SWE after the Fontan. Demonstrating an association between hemodynamics and SWE may allow for individualizing the frequency of post-Fontan FALD surveillance.

As previously reported, our study found higher SWE in Fontan patients, with no significant correlation of abnormal liver stiffness with unfavorable hemodynamics during pre- or post-Fontan cardiac catheterization, systemic ventricular dysfunction, or severity of atrioventricular valve regurgitation. While this may seem counterintuitive, we believe this data is vital in highlighting that significantly elevated liver stiffness may occur in patients without evidence of adverse hemodynamics or high-risk echocardiographic findings.

Our study has limitations, including its single-center, retrospective nature, younger population, and small sample size, which limits our ability to detect subtle associations. Additionally, US elastography can be operator dependent, and selecting the correct probe is the key to ensuring appropriate sampling of liver tissue, which can be challenging in children. However, at our center, these studies are performed by experienced ultrasound technicians in radiology using robust quality control measures to maintain high diagnostic accuracy.

Furthermore, as this was a retrospective study, the time of cardiac catheterization and echocardiogram were not standardized with the time of US elastography evaluation. Longitudinal studies with standardized protocols for using elastography in Fontan patients are warranted.

## 5 Conclusion

Liver US elastography is a widely used non-invasive diagnostic tool in surveillance for FALD. Typical SWE velocities are higher than usual in the Fontan population. In our study, SWE did not correlate with adverse hemodynamics before or after Fontan operation. Further, there was no correlation with significant AV valve regurgitation or ventricular dysfunction. Our findings further highlight the need for a standardized approach for surveillance in Fontan patients to better assess chronic Fontan-related complications while pursuing the quest for an ideal non-invasive tool for FALD.

## Figures and Tables

**Table 1:** Characteristics of patients with Fontan operation.

Variable	*N* = 56(%)
**Male, *n* (%)**	32 (57.1)
**White, *n* (%)**	42 (75)
**Median Age at liver US elastography, years [IQR]**	12.5 [9, 16.75]
**Physiology, *n* (%)**	
Single left ventricle	19 (33.9)
Single right ventricle	37 (66.1)
**Primary diagnosis, *n* (%)**	
HLHS	25 (44.6)
Tricuspid atresia	8 (14.3)
UAVSD	6 (10.7)
PA/IVS	5 (8.9)
Other complex anatomy	5 (8.9)
DILV	4 (7.1)
DORV	2 (3.6)
Congenitally corrected TGA	1 (1.8)
**Heterotaxy, *n* (%)**	
No	50 (89.3)
Right atrial isomerism	4 (7.1)
Left atrial isomerism	2 (3.6%)
**Type of Fontan, *n* (%)**	
Extracardiac	44 (78.6)
Lateral tunnel	10 (17.9)
Atriopulmonary connection	2 (3.6)
**Fenestration, *n* (%)**	
No	31 (55.4)
Yes	25 (44.6)
Currently patent	15 (60)
Spontaneously closed/not well seen	3 (12)
Closed by intervention	7 (28)
**Other Fontan-related comorbidities, *n* (%)**	
Atrial arrhythmias	21 (55.3)
Thrombocytopenia	16 (42.1)
Esophageal varices	6 (15.8)
Serious thromboembolic event	6 (15.8)
Protein-losing enteropathy	5 (13.2)
AV block	3 (7.9)
Pacemaker	3 (7.9)
Sinus node dysfunction	1 (2.6)
Plastic bronchitis	1 (2.6)
Ventricular arrhythmia	1 (2.6)
**Transplant, *n* (%)**	
Isolated heart	10 (17.8)
Combined heart/liver	1 (1.8)

DILV: double inlet left ventricle, DORV: double outlet right ventricle, HLHS: hypoplastic left heart syndrome, PA/IVS: pulmonary atresia intact ventricular septum, UAVSD: unbalanced atrioventricular septal defect.

**Table 2:** SWE as a function of time (years) from Fontan operation.

Years after Fontan	Total	SWE Median (m/s)
<5 years	21 (26.9%)	1.85 [1.44–2.00]
5–10 years	24 (30.7%)	1.91 [1.64–1.99]
>10 years	33 (42.3%)	1.94 [1.44–2.04]

**Table 3:** Correlation between cardiac catheterization data and SWE (analyzed as continuous variables).

Pre-Fontan	Median [IQR]	*p* Value
Qep (L/min/m^2^)	2.45 [2.21, 3.16]	0.46
Ventricular end-diastolic pressure (mmHg)	10 [8, 10.5]	0.77
Glenn pressure (mmHg)	12 [13, 15]	0.83
Post-Fontan	Median [IQR]	*p* Value
Qs (L/min/m^2^)	2.80 [2.4, 3.34]	0.93
Ventricular end-diastolic pressure (mmHg)	12 [13.5, 14]	0.99
Fontan pressure (mmHg)	18 [16, 21]	0.86
Systemic saturation (%)	93 [87, 96]	0.77
Indexed pulmonary vascular resistance (WU·m^2^)	1.80 [1.49, 2.37]	0.93

Qep: effective pulmonary flow, Qs: systemic flow.

**Table 4:** Correlation between cardiac catheterization data and SWE (analyzed as a dichotomized variable).

Pre-Fontan	SWE Median (m/s)	*p* Value
Qep ≤ 2	1.96 [1.67, 2.04]	0.96
Qep > 2	1.96 [1.66, 2.0]	
Ventricular end-diastolic pressure ≤ 12	1.9 [1.68, 2.0]	0.71
Ventricular end-diastolic pressure > 12	1.96 [NA]	
Glenn pressure ≤ 15	1.96 [1.6, 2.0]	0.95
Glenn pressure > 15	1.9 [1.63, 2.04]	
Post-Fontan	SWE Median (m/s)	*p* Value
Qs ≤ 2	2.01 [NA]	0.71
Qs > 2	1.96 [1.66, 2.0]	
Ventricular end-diastolic pressure ≤ 12	1.80 [1.73, 2.04]	0.27
Ventricular end-diastolic pressure > 12	1.98 [1.39, 2.3]	
Fontan pressure ≤ 15	2.04 [1.79, 2.55]	0.109
Fontan pressure > 15	1.96 [1.66, 2.01]	
Systemic saturation ≤ 90	2 [1.52, 2.42]	0.50
Systemic saturation > 90	1.96 [1.69, 2.01]	
Indexed pulmonary vascular resistance ≤ 2.5	1.97 [1.73, 2.04]	0.25
Indexed pulmonary vascular resistance > 2.5	1.74 [1.39, 2.35]	

Qep: effective pulmonary flow, Qs: systemic flow. NA: not enough patients.

**Table 5:** Correlation between echocardiographic data and SWE (analyzed as a dichotomized variable).

Systemic Ventricular Systolic Function	SWE Median (m/s)	*p* Value
Better than moderate	1.97 [1.69, 2.01]	0.35
Moderate or worse	1.67 [1.3, 2.19]	
Systemic Atrioventricular Valve Regurgitation	SWE Median (m/s)	*p* Value
Better than moderate	1.96 [1.54, 2.01]	0.35
Moderate or worse	1.9 [1.67, 2.1]	
